# Supplementary material for: Identification and validation of key genes associated with pathogenesis and prognosis of gastric cancer
Source: PeerJ. 2023 Oct 16;11:e16243. doi: 10.7717/peerj.16243 (PMC10586292; doi:10.7717/peerj.16243)
Supplement: Supplemental Information 1 [file peerj-11-16243-s001.docx]

| **RUN** | **BioSample** | **Library Name** | **Tissue Subtype** |
| --- | --- | --- | --- |
| SRR17278707 | SAMN24247315 | GSM5742871 | Gastric cancer |
| SRR17278708 | SAMN24247316 | GSM5742870 | Gastric cancer |
| SRR17278709 | SAMN24247317 | GSM5742869 | Gastric cancer |
| SRR17278710 | SAMN24247318 | GSM5742868 | Gastric cancer |
| SRR17278711 | SAMN24247319 | GSM5742867 | Gastric cancer |
| SRR17278712 | SAMN24247320 | GSM5742866 | Gastric cancer |
| SRR17278713 | SAMN24247321 | GSM5742865 | Gastric cancer |
| SRR17278714 | SAMN24247322 | GSM5742864 | Gastric cancer |
| SRR17278715 | SAMN24247323 | GSM5742863 | Intestinal metaplasia |
| SRR17278716 | SAMN24247324 | GSM5742862 | Intestinal metaplasia |
| SRR17278717 | SAMN24247325 | GSM5742861 | Intestinal metaplasia |
| SRR17278718 | SAMN24247326 | GSM5742860 | Intestinal metaplasia |
| SRR17278719 | SAMN24247327 | GSM5742859 | Intestinal metaplasia |
| SRR17278720 | SAMN24247328 | GSM5742858 | Intestinal metaplasia |
| SRR17278721 | SAMN24247313 | GSM5742873 | Gastric cancer |
| SRR17278722 | SAMN24247314 | GSM5742872 | Gastric cancer |
| SRR17278723 | SAMN24247330 | GSM5742856 | Intestinal metaplasia |
| SRR17278724 | SAMN24247332 | GSM5742854 | Intestinal metaplasia |
| SRR17278725 | SAMN24247333 | GSM5742853 | Non-atrophic gastritis |
| SRR17278726 | SAMN24247331 | GSM5742855 | Intestinal metaplasia |
| SRR17278727 | SAMN24247329 | GSM5742857 | Intestinal metaplasia |
| SRR17278728 | SAMN24247334 | GSM5742852 | Non-atrophic gastritis |
| SRR17278729 | SAMN24247335 | GSM5742851 | Non-atrophic gastritis |
| SRR17278730 | SAMN24247337 | GSM5742849 | Non-atrophic gastritis |
| SRR17278731 | SAMN24247339 | GSM5742847 | Non-atrophic gastritis |
| SRR17278732 | SAMN24247340 | GSM5742846 | Non-atrophic gastritis |
| SRR17278733 | SAMN24247338 | GSM5742848 | Non-atrophic gastritis |
| SRR17278734 | SAMN24247336 | GSM5742850 | Non-atrophic gastritis |
| SRR17278735 | SAMN24247341 | GSM5742845 | Non-atrophic gastritis |
| SRR17278736 | SAMN24247342 | GSM5742844 | Non-atrophic gastritis |
